# Frequency of complications after dermatological surgeries in the elderly^☆^

**DOI:** 10.1016/j.abd.2021.03.015

**Published:** 2022-07-15

**Authors:** Isabella Parente Almeida, Maria Isabel Ramos Saraiva, Maria Cristina de Lorenzo Messina, Luiz Guilherme Martins Castro

**Affiliations:** Private Dermatology Clinic, São Paulo, SP, Brazil; b Hospital Alemão Osvaldo Cruz, Cutaneous Oncology Sector, São Paulo, SP, Brazil; c Hospital Israelita Albert Einstein, Cutaneous Oncology Sector, São Paulo, SP, Brazil; d Hospital Ipiranga, Department of Dermatology, São Paulo, SP, Brazil

Dear Editor,

Population aging is a reality in Brazil. Both the incidence and prevalence of skin cancer are higher among the elderly.^1^ This explains the growing demand by the elderly for skin cancer treatments.^1^ Contrary to what many believe, dermatological surgeries (DS) in the elderly do not pose a higher risk of complications than in young individuals.^2^, ^3^ Yet, there is some resistance by dermatologists to indicate surgery as the first-choice procedure for this population.

This study was developed to assess the risk of complications after DS was performed in the elderly population, aiming to evaluate and compare the postoperative complication rate (POCR) after DS in patients from three advanced age groups.

A retrospective, single-center study was carried out in a private service. A review of electronic medical records of patients over 65 years of age, operated on a day-hospital regimen, was performed between August 2012 and July 2018. The patients were divided into three groups, according to their age on the day of surgery. The elderly group (E) involved patients aged 65 to 74 years; patients aged 75 to 84 years were included in the very elderly (VE) group and patients aged 85 years and over were allocated to the extremely elderly (EE) group.

For patients submitted to more than one surgical procedure throughout the study, data from each intervention were computed separately. When multiple lesions were operated on during the same intervention, each one was computed separately aiming to calculate POCR (number of lesions that showed complications/number of lesions operated on). The postoperative complications (POCs) were classified into 4 types: dehiscence, hemorrhage, necrosis, and infection. Almost all procedures were performed under local anesthesia or local anesthesia and sedation.

The data were analyzed using the BioEstat 5.3 program (Brazil), with a significance level of p ≤ 0.05 for all tests. Pearson’s Chi-Square and Fisher’s Exact tests were used for comparison between the groups.

Detailed results are shown in Table 1. Regarding the POCR, there were no statistically significant differences between the groups (p > 0.05; E×VE p = 0.308; VE×EE p = 0.6832; E×EE p = 0.1798). The group with the lowest POCR was the EE (6.0%), albeit without statistical significance.

**Table 1:** Epidemiological and clinical characteristics and post-surgical complications according to the age group.

	Elderly (65‒74 years)	Very elderly (75‒84 years)	Extremely elderly (85 or + years)
**Number of patients**	74	76	55
**Sex (F/M)**	29/57	24/84	35/46
**Mean age (years)**	70,2	76,8	88,8
**Median age (years)**	70	80	88
**Number of addressed lesions**	163	243	199
**Number of surgical procedures**	86	108	81
**Number of lesions with complications (%)**	16 (9,8%)	17 (7,0%)	12 (6,0%)
**Diagnosis:**			
BCC (%)	72 (44,2%)	107 (44%)	74 (37,2%)
SCC (%)	23 (14,1%)	53 (21,8%)	56 (28,1%)
Melanoma (%)	9 (5,5%)	7 (2,9%)	2 (1,0%)
Others	59 (36,2%)	76 (31,3%)	67 (33,7%)
**Lesion location:**			
Head and neck	59 (36,2%)	115 (47,3%)	98 (49,2%)
Trunk	62 (38,0%)	71 (29,2%)	53 (26,6%)
UL	15 (9,2%)	21 (8,7%)	14 (7,1%)
LL	27 (16,6%)	36 (14,8%)	34 (17,1%)

SAH, Systemic Arterial Hypertension; DM, Diabetes Mellitus; BCC, Basal Cell Carcinoma; SCC, Squamous Cell Carcinoma; UL, Upper Limbs; LL, Lower Limbs.

Comparing the POCR in relation to the surgical site, regardless of the age group, a higher percentage of complications was observed on the lower limbs (LL) surgeries (16.5%) and a lower percentage on the upper limbs (UL) surgeries (4.0%; p = 0.033). LL surgeries also showed more complications than those on the trunk (p = 0.007237) and head and neck (HN; p = 0.000817). There was no statistical difference between POCR involving UL, trunk, and HN when compared two by two (Fig. 1).

**Figure 1:**
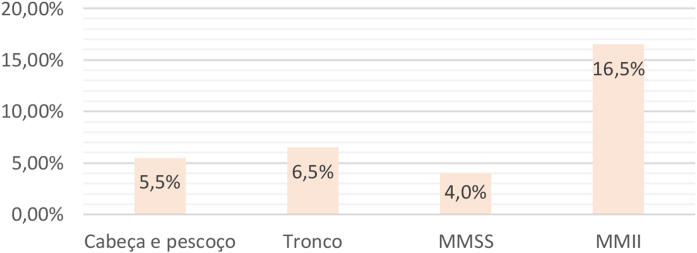
Postoperative complication rates according to the operated body segment, regardless of the age group. UL, Upper Limbs; LL, Lower Limbs.

Among the patients in group I who had complications, the mean age was 70.1 years. The most common complication was surgical wound dehiscence, which occurred in 44.4% of the lesions that showed complications. Half of the lesions that progressed to POC were located on the trunk.

Of the 243 lesions excised on the LL of the patients, 17 (6.7%) developed some complications (Table 2). The mean age of the LL patients who showed complications was 79.4 years. Dehiscence was also the most common complication, observed in 36.8% of the lesions that had complications. The LL was the most affected site by POC (41.2%) in this group.

**Table 2:** Epidemiological, clinical and surgical characteristics of patients with postoperative complications, according to the age group.

	Elderly (65-74 years)	Very elderly (75-84 years)	Extremely elderly (85 or + years)	Total
**Sex:**				
Male	13 (81.3%)	14 (82.4%)	7 (58.3%)	34 (75.6%)
Female	3 (19.7%)	3 (17.6%)	5 (41.7%)	11 (24.4%)
**Mean age:**	70.1 years	79.4 years	88.5 years	
**Median age:**	70.0 years	79.0 years	87.5 years	
**Comorbidities:**				
SAH	11 (68.8%)	14 (82.4%)	11 (91.7%)	36 (80.0%)
DM	9 (56.3%)	7 (41.2%)	3 (25.0%)	19 (42.2%)
Pacemaker	0	1 (5.9%)	3 (25.0%)	4 (8.9%)
**Type of complication:**				
Necrosis	3 (18.8%)	3 (17.6%)	1 (8.3%)	7 (15.6%)
Dehiscence	8 (50.0%)	8 (47.1%)	4 (33.3%)	20 (44.4%)
Hemorrhage	3 (18.8%)	1 (5.9%)	1 (8.3%)	5 (11.1%)
Infection	3 (18.8%)	7 (41.2%)	5 (41.7%)	15 (33.3%)
Other	1 (5.6%)	0	2 (6.7%)	3 (6.7%)
**Location of complication:**				
Head and neck	5 (31.3%)	6 (35.3%)	4 (33.33%)	15 (33.3%)
Trunk	8 (50%)	3 (17.7%)	1 (8.33%)	12 (26.7%)
UL	0	1 (5.9%)	1 (8.33%)	2 (4.4%)
LL	3 (18.7%)	7 (41.2%)	6 (50%)	16 (35.6%)

Of the 199 lesions excised on in EE patients, 12 (6.0%) showed complications (Table 2). The mean age of the EE group was 88.5 years. Infection was the most common POC (38.5%) and the LL was the most affected site by POC (50%).

There was no statistically significant difference between POCR in age groups from 65 years old onward. These data go against the simplistic view that elderly patients would have a higher risk of complications, leading dermatologists to give up the surgical option for these patients. Regarding the POCR by age group, the values found in the present investigation are similar to those observed in other studies involving elderly individuals, where these rates ranged from 5.7% to 10.6%.^2^, ^3^, ^4^

Changes resulting from aging and the presence of comorbidities in the elderly would place this group at a greater risk for POC. However, Imamura et al. demonstrated that in Japanese patients the POCR after DS was similar between the elderly aged between 75 and 80 years and those aged over 90 years.^3^ The finding that POCR did not increase with advancing age has been demonstrated in different studies, which showed that performing DS in the “extremely elderly” is as safe as in other elderly individuals.^2^, ^3^, ^4^

Regarding the risk of complications per operated body segment, it was observed that lesions on the lower limbs show more complications than those on the trunk or HN. The findings of the present study differ from those described by Paredela et al, who found no correlation between the excised site and complications.^4^ On the other hand, O’Neill et al. observed lower POCR in lesions located on the face.^5^ There is no consensual explanation about the reasons that lead to a higher risk of complications on the lower limbs. The authors suggest that the venous return impairment, which is frequently observed in the elderly population, combined with skin changes resulting from chronic venous stasis would contribute to higher POCR.

The lack of analysis of the surgical complexity, since more complex surgeries would have a greater chance of developing POC, is a possible study bias. The number of excised lesions in the same surgical procedure was not considered either, which also could interfere with the postoperative evolution. The use of certain medications, such as anticoagulants, antiplatelet agents, and the existence of comorbidities can interfere with the POCR. An additional study is being performed to assess the influence of these variables.

The POCR after DS did not vary significantly among three age groups of incresingly elderly individuals. Surgeries on the lower limbs showed higher POCR than those performed on other body segments, regardless of the assessed age group.

## Financial support

None declared.

## Authors’ contributions

Isabella Parente Almeida: Statistical analysis; design and planning of the study; drafting and editing of the manuscript; collection, analysis, and interpretation of data; critical review of the literature.

Maria Isabel Ramos Saraiva: Statistical analysis; design and planning of the study; drafting and editing of the manuscript; collection, analysis, and interpretation of data; critical review of the literature.

Maria Cristina de Lorenzo Messina: Approval of the final version of the manuscript; design and planning of the study; collection, analysis, and interpretation of data; intellectual participation in the propaedeutic and/or therapeutic conduct of the studied cases; critical review of the manuscript.

Luiz Guilherme Martins Castro: Approval of the final version of the manuscript; design and planning of the study; collection, analysis, and interpretation of data; effective participation in research orientation; intellectual participation in the propaedeutic and/or therapeutic conduct of the studied cases; critical review of the manuscript.

## Conflicts of interest

None declared.
